# A Data‐Driven Approach Towards Understanding the Volume and Complexity of Young Adults With Chronic Medical Conditions Transitioning to Adult Healthcare: A Cross‐Sectional Study

**DOI:** 10.1111/jpc.70508

**Published:** 2026-07-21

**Authors:** Mary White, Ameer Lambrias, Evelyn Culnane, Alice Voskoboynik

**Affiliations:** ^1^ Health Services & Economics Murdoch Children's Research Institute Parkville Victoria Australia; ^2^ Department of Endocrinology & Diabetes The Royal Children's Hospital Parkville Victoria Australia; ^3^ Melbourne School of Population and Global Health University of Melbourne Carlton Victoria Australia; ^4^ Department of Paediatrics University of Melbourne Melbourne Australia; ^5^ Transition Support Service The Royal Children's Hospital Parkville Victoria Australia; ^6^ Centre for Health Analytics The Royal Children's Hospital Parkville Victoria Australia

## Abstract

**Aims:**

To describe the volume, complexity, referral patterns and pre‐transition supports provided to a cohort of adolescents/young adults (AYAs) transitioning to adult healthcare services from a tertiary paediatric centre.

**Methods:**

This is a cross‐sectional retrospective review of AYAs aged ≥ 17 years who attended their *final* paediatric clinic review 1 January 2017–31 December 2018 with one or more of the most common chronic medical conditions or who attended the most frequently attended medical clinics at The Royal Children's Hospital, Melbourne.

**Results:**

A total of 592 AYA (female = 283, 47.8%) met the inclusion criteria, with a mean (SD) age at the final RCH clinic of 18.6 (1.1) years. Of these, 115 (19.43%) were discharged to primary care, 44 (7.43%) disengaged from RCH prior to formal transition 44 (7.43%) and 3 (1.85%) died prior to completion of the transition process. Of the 430 AYA who were referred for ongoing specialist care, 384 (89.3%) were expected to be reviewed within the public system. Pre‐transition support was accessed by 106/430 (24.65%) where 83 (19.3%) received standardised support. Higher representation in those who received coordinated care (*n* = 23, 5.35%) was seen in those; with ≥ 2 ongoing conditions, from regional postcodes, requiring interpreter assistance, with documented intellectual disability; and specific conditions such as epilepsy, inflammatory bowel disease and cerebral palsy.

**Conclusions:**

Referral patterns and the nature of transition support at our institution are clinician/department dependent and therefore access may not be equitable between or even within condition groups. These data provide a platform from which robust post‐transition outcome data and policy can be generated.

## Key Points

- What is already known.
  ○ Transition to adult care is known to be a time of vulnerability for young adults with chronic and complex medical conditions.
  ○ Robust policy beyond generic recommendations is required in this area, limited by the dearth of rigorous research studies and longitudinal data.
  ○ Mechanisms to identify those AYA who are at highest risk are also lacking which restricts the potential to allow direction of limited resources to the most vulnerable.
- What this paper adds.
  ○ Comprehensive clinical and demographic description of a transitioning cohort of AYA from the largest paediatric referral centre in Victoria.
  ○ Assessment of reach and nature of transition support services in the context of complexity.

## 1 Background

Transition to adult care is well‐recognised as a time of vulnerability for adolescents and young adults (AYAs) with chronic medical conditions (CMCs) [1]. An estimated 2.5 million AYAs with CMCs will need to transfer from paediatric to adult care over the next 20 years in Australia [2, 3]. Siloed healthcare services and a lack of accessible data‐linkage infrastructure hinder the systematic capture of pre‐ or post‐transition data at scale beyond existing condition or clinic‐specific healthcare utilisation reports [4, 5]. Whilst successful transition has yet to be defined, clinicians are aware that unsuccessful transition can have profound consequences. It has been demonstrated that suboptimal experiences can lead to an increase in unplanned or crisis‐driven care (increased hospital admissions and emergency room visits [6, 7]) and a decrease in routine and preventative outpatient care [8].

The Royal Australian College of Physicians recommends that ‘All young people with a chronic illness or disability should have a health care provider who takes specific responsibility for their transition to adult health care’, care which includes service coordination and the provision of written transition plans [1], yet for time poor clinical teams this is not always feasible. When available, specialised paediatric transition support services aim to assume this role, preparing AYAs for the practical and emotional challenges of transition to adult care. In Australia, funding for these important clinical adjuncts varies across states and healthcare services. The Royal Children's Hospital, Melbourne (RCH) is a stand‐alone paediatric hospital and is the largest tertiary paediatric hospital in Victoria. The *Transition Support Service* has been available at RCH since 2010. Support ranges from standardised pre‐transition counselling, through to complex care coordination across multiple sites and services. The principles which inform the care that the service provides includes; ensuring high quality post‐transition health care which is appropriate for the young person's age, culture and development; providing a flexible process which is responsive and relevant to individual needs; provision of support to develop skills in communication, decision‐making, assertiveness and self‐care and self‐advocacy; improving the young person's independence with their health care as appropriate and to the best of their ability; provision of support and guidance for parents/carers; maximising the young person's capabilities to live well and achieve their goals regardless of their condition or disability. The proportion of transitioning AYA who access this service at RCH is not currently known, nor whether access is equitable across departments or conditions. Given the resource intense nature of this service in a system where resources are in turn limited, prioritisation of transitioning AYAs in terms of complexity or need may be a useful tool in terms of differentiating those who would benefit most from a more tailored approach to supportive transition care. Complexity in this setting has not formally been defined or assessed previously, although assessment of complexity based on medical and psychosocial factors has been validated in other settings [9, 10].

Despite tacit knowledge that systemic issues exist, we have limited understanding of how or if the Australian healthcare system is meeting the needs of AYA with CMCs. From a paediatric perspective, robust data to support transition service planning is essential to formally define the magnitude of the clinical cohort and the multiplicity of their needs; and where available, access to transition support services should be equitable and appropriate to the level of need.

## 2 Aims

The specific aims were to:

1. Identify the number of young adults who transitioned from our paediatric healthcare service between 1 January 2017 and 31 December 2018 with one or more of the most common chronic medical conditions/condition categories or who attended one or more of the most frequently attended outpatient clinic services.
2. Describe the referral patterns to adult services (public or private) or primary care and whether these ongoing referrals were supported by the provision of a documented formal transfer letter.
3. Describe the relative complexity of this cohort in terms of the number of co‐existing medical conditions, presence of documented mental health issues, language and interpreter requirements, SES, history of vulnerability (as denoted by annotations on the electronic medical record (EMR) indicating a history of family violence or other psychosocial complexity) and using a novel scoring system.
4. Identify the proportion of AYA who were counselled by the Transition Service team to describe the level of support provided (none, standard, care coordination), patterns of uptake of this service based on medical condition complexity and postcode of primary residence at the time of transition

### 2.1 Methods

In order to refine the participant characteristics for this retrospective cohort study, an electronic medical record (EMR, EPIC [11]) search to determine the most common medical conditions and most commonly attended outpatient services for young adults aged ≥ 17 between 1 January 2017 and 31 December 2018 was initially conducted. This search identified that the most common chronic medical conditions experienced by AYA who were most likely to transition from paediatric care within the study period were Type 1 diabetes mellitus, epilepsy, inflammatory bowel disease, congenital heart disease, cerebral palsy, anorexia nervosa, cystic fibrosis and chronic renal insufficiency. The most frequently attended clinics in this age‐group were general medicine, gastroenterology, neurology, endocrinology, cardiology, respiratory medicine, nephrology, developmental medicine, adolescent medicine and orthopaedics. Combined this accounted for 90.1% (2539/2796) of AYAs who met the age and date criteria. Thereafter, a targeted EMR data extraction was conducted in September 2023 of all encounters with young adults aged ≥ 17 years who had their *final* paediatric clinic review at RCH over the stated 2‐year period for one or more of the aforementioned clinical conditions or clinics. For each individual, the following data was accessed; the date of the last RCH outpatient clinic (date of transfer), age at transfer to adult services, the predominant medical condition/team as per the primary or underlying diagnosis or condition, preferred language/interpreter requirements, the presence of any vulnerability flags within the EMR and postcode as a proxy to SEIFA. A subsequent manual review of each medical record was conducted January–September 2024 in order to document; instances where disengagement occurred prior to formal transfer to adult service care, the presence/absence of a formal transition referral letter; the centre(s)/setting of referral (documented from a transition referral letter or stated within the clinical notes); documented mental health diagnoses, behavioural concerns, intellectual disability or learning issues; and the nature of the involvement with the Transition Support Service. ‘Standard’ transition care was defined as support provided according to standardised education sessions around engaging with the healthcare system and their medical care (accessing a Medicare card, awareness of condition and medications etc.) where ‘coordinated’ care was denoted when the Transition support team were additionally involved with appointment or management coordination with external providers such as the adult teams or community supports, including participation in joint clinics with the paediatric and adult medical teams. For the purposes of this study, where there was no documented interaction with the Transition Support Service or where generation of a transition passport within the EMR (documentation of the transition pathway) was the only contact with the team without any direct contact with the AYA or their carers, this was denoted as ‘no support’. The resources utilised and provided by the RCH Transition service can be accessed via their website [12]. Data were excluded where the individual was discharged after a single paediatric review at RCH.

Child socioeconomic status (SES) was derived from the patient residential postcode using the Australian Bureau of Statistics (ABS) Socio‐Economic Indexes for Areas (SEIFA) and its derived Index of Relative Socio‐economic Disadvantage (IRSD) [13] where lower scores correspond to greater disadvantage. The IRSD was categorised according to IRSD deciles where ‘Lower SES’ incorporated IRSD decile areas 1–5. Remoteness was defined based on the Remoteness Areas Structure within the Australian statistical geography standard (ASGS) of the ABS [8], dividing Australia into five classes of remoteness based on a measure of relative access to services: major cities, inner regional, outer regional, remote and very remote. Inner regional, outer regional, remote and very remote areas were aggregated and classified as regional Victoria, which covers all areas of Victoria, except metropolitan Melbourne. Participant transition complexity was denoted as per a novel complexity scoring assessment whereby an additional point was allocated based on each of the following: two or more ongoing conditions requiring specialist care, the presence of an interpreter flag, low SEIFA (IRSAD deciles 1–5), vulnerability flag, indigenous flag, documented intellectual disability, documented neurodevelopmental condition or a documented mental health condition. The characteristics of individuals exposed to transition supports were compared using ANOVA and chi‐square analyses for continuous and categorical variables respectively. Organisational approval from the RCH HREC was obtained prior to commencement of this Quality Assurance project (QA/85357/RCHM‐2022).

## 3 Results

Initially, 599 individuals were identified on the initial EMR search (female = 285), 7 of whom were removed from the dataset after a single paediatric encounter (autism: *n* = 2; depression: *n* = 1; migraine: *n* = 1; well adolescent: *n* = 1; neurogenic bladder: *n* = 1). Therefore total of 592 AYA (female = 283, 47.8%) met the inclusion criteria for the study, with a mean (SD) age at the final RCH clinic of 18.6 (1.1) years. Of these, 115 (19.43%) young adults were discharged to primary care for ongoing follow up, 44 (7.43%) disengaged from RCH prior to formal transition/transfer planning 44 (7.43%) and 3 (1.85%) died prior to completion of the transition process. Therefore, a total of 430 AYA were included in this analysis, 384 (89.3%) and 46 (10.7%) of whom were expected to be reviewed within the to a public and private systems respectively for ongoing care. A referral letter was documented within the medical record for 379 (88.76%) of AYA. Pre‐transition support via the RCH Transition Service was accessed by 106/430 (24.65%) AYA where standardised support was provided to 83 (19.3%) of AYA and coordinated care was provided to 23 (5.35%) of the cohort, Table 1. As per the transition complexity scoring approach, 258 (60%) were assigned a score ≥ 1, where 85 (19.77%) AYA were assigned a score of ≥ 2.

**TABLE 1:** Demographics, clinical features, referral data and transition support service data.

			Pre‐transition support			*p*
Expected to transition to ongoing specialist adult care		*n* = 430	None^a^, *n* = 324 (75.35)	Standard, *n* = 83 (19.3)	Coordinated care, *n* = 23 (5.35)	
Age at final paediatric review, *n* (%)		18.58 (0.69)	18.55 (1.14)	18.63 (0.59)	18.5 (0.81)	0.5307
Sex	Female	201 (46.74)	155 (47.84)	37 (44.58)	9 (39.13)	0.875
	Male	228 (53.02)	168 (51.85)	46 (55.42)	14 (60.87)	
	Other	1 (0.23)	1 (0.31)	0 (−)	0 (−)	
Setting of expected ongoing care	Public adult service	384 (89.3)	288 (88.89)	75 (90.36)	21 (91.3)	0.882
	Private adult specialist	46 (10.7)	36 (11.11)	8 (9.64)	2 (8.7)	
Referral letter provided for specialist care, *n* (%)		379 (88.76)	286 (88.82)	75 (90.36)	18 (81.82)	0.528
Interpreter requirement, *n* (%)		8 (1.86)	4 (1.23)	2 (2.41)	2 (8.7)	0.035
Vulnerability flag, *n* (%)		3 (0.7)	3 (0.93)	0 (−)	0 (−)	0.61
Indigenous flag, *n* (%)		3 (0.74)	1 (0.33)	2 (2.47)	0 (−)	0.124
Low SEIFA^b^, *n* (%)		162 (37.67)	123 (37.96)	29 (34.94)	10 (43.48)	0.739
Rurality, *n* (%)	Metro	341 (79.3)	257 (79.32)	68 (81.93)	16 (69.57)	0.432
	Regional	89 (20.7)	67 (20.68)	15 (18.07)	7 (30.43)	
Principal condition, *n* (%)	T1DM	172 (40)	130 (40.12)	42 (50.6)	0 (−)	0.000
	Congenital heart disease	76 (17.67)	73 (22.53)	1 (1.2)	2 (8.7)	
	Epilepsy	74 (17.21)	61 (18.83)	3 (3.61)	10 (43.48)	
	Inflammatory bowel disease	44 (10.23)	18 (5.56)	23 (27.71)	3 (13.04)	
	Cerebral palsy	22 (5.12)	13 (4.01)	5 (6.02)	4 (17.39)	
	Eating disorder	8 (1.86)	8 (2.47)	0 (−)	0 (−)	
	Cystic fibrosis	7 (1.63)	0 (−)	6 (7.23)	1 (4.35)	
	Chronic kidney disease	7 (1.63)	3 (0.93)	3 (3.61)	1 (4.35)	
	Other^c^	10 (2.33)	9 (2.78)	0 (−)	0 (−)	
Documented intellectual disability, *n* (%)		27 (6.28)	15 (4.63)	5 (6.02)	7 (30.43)	0.000
Documented neurodiversity, *n* (%)		29 (6.97)	23 (7.42)	3 (3.61)	3 (13.04)	0.241
Documented mental health condition, *n* (%)		62 (14.45)	46 (14.24)	10 (12.05)	6 (26.09)	0.232
Number with two or more healthcare conditions at the time of final paediatric review, *n* (%)		114 (26.51)	75 (23.15)	28 (33.73)	11 (47.83)	0.009
Transition complexity score of 2 or higher^d^		64 (14.88)	42 (12.96)	14 (16.87)	8 (34.78)	0.015

*Note:* Between group differences were assessed using one‐way ANOVA and chi‐squared analyses for continuous and categorical variables, respectively.

^a^ None—denotes zero contact with the transition support team OR development of a passport in the EMR as the only interaction with the Transition Support Service at RCH.

^b^ Low SEIFA denotes deciles 1–5 as calculated from postcode at the time of transition.

^c^ Other relates to complex genetic condition (*n* = 4), non‐brain malignancy(*n* = 3), brain tumour (*n* = 2), non‐diabetes endocrinology (*n* = 1), gender dysphoria (*n* = 1), liver disease (*n* = 1).

^d^ As per the proposed complexity scoring assessment whereby an additional point was allocated based on each of; two or more ongoing conditions requiring specialist care, the presence of an interpreter flag, low SEIFA, presence of a vulnerability flag, indigenous flag, documented intellectual disability, documented neurodevelopmental condition or a documented mental health condition.

Variations in referral patterns to the Transition Service were seen across condition groups and according to AYA characteristics. Higher representation in those who received coordinated care was seen in AYAs with two or more ongoing conditions, complexity scores ≥ 2, from regional postcodes, requiring interpreter assistance, with documented intellectual disability and specific conditions such as epilepsy, inflammatory bowel disease and cerebral palsy.

## 4 Discussion/Conclusion

This cohort study provides context to describe the volume, referral practises and complexity of young adults with CMCs at the time of transition from a tertiary paediatric healthcare service. The key findings highlight that the majority of AYA in the cohort were expected to be seen within adult public healthcare services and approximately 25% of those referred for ongoing specialist care received pre‐transition counselling/support from the specialised Transition Support Service. Appropriate disproportional representation of more high need conditions/states was seen in the care coordination group. Almost 90% had a documented transfer letter accessible in EMR which, whilst suboptimal, reflects a significant change in practise compared to historical RCH data [14]. Approximately 15% of the cohort had a complexity score of ≥ 2 denoting medical or psychosocial risk or both. There are no equivalent Australian or international multi‐condition datasets on which to directly compare these data.

Transition support services at RCH and elsewhere are resource limited (current staff EFT is 2.4) and it is not feasible to deliver one‐on‐one support to all AYAs in the lead up to transition. Referral patterns and the nature of involvement at our institution are clinician/department dependent (where joint transition clinics are provided to some but not all conditions), and therefore access may not be equitable between or even within condition groups. The service has to prioritise those who are transitioning into specialist care, which means that important personal or generic advice (including prescription management, Medicare card function and the nature of healthcare services) is lacking for those discharged back to primary care and indeed AYAs who do not receive this support. The proportion of patients seen through the RCH Transition service has increased over time yet further scaling up of the Transition Service would require a significant increase in resources. The number of joint clinics has also increased across all the subspecialty groups since 2018 and bespoke joint clinics, including general practitioners as well as adult specialists, are now available for the most complex patients.

This is a comprehensive dataset from one of the largest standalone paediatric hospitals in the Southern Hemisphere. Participants are well characterised in terms of complexity and access to a well‐established transition support service. However, these data are derived from a single centre where the majority of participants were noted to be from higher SEIFA metropolitan areas, with low numbers from documented CALD and indigenous communities. From a feasibility perspective, the conditions included in this study were limited to those most commonly documented, representing 90% of all AYA who were last seen at RCH within the parameters of the study period. The complexity score described herein is novel and the utility of this is untested at this time but there are plans to validate its utility with regards to post‐transition outcomes in ongoing work. The data included in this study is limited to that which could be retrieved from the medical records similar to approaches used elsewhere [10]. Since documentation of important aspects of well‐being for example, health related quality if life, exposure to adverse childhood events or clinical status are not recorded formally, this information could not be included. However, these data can be derived from administrative data/EMR data, which is an important consideration should systematic use be planned in future work. Documentation of intellectual disability, mental health issues, neurodiversity, vulnerability is clinician dependent and therefore is likely to be incomplete. For example, reported rates of mental health concerns significantly lower than what might be expected in a cohort of AYA with CMC [15]. Introduction of a system where families or young adults could add these data to the medical record would simultaneously increase the quality of the data recorded in the EMR and would also provide clinicians with opportunities to explore whether additional supports are needed. There are no existing Australian data which document clinical outcomes beyond 2 years post‐transfer from paediatric care which impedes opportunities to lobby government or to develop substantial policy change beyond generic principles. These data were collected and recorded for participants who transitioned to adult care in 2017/2018. Whilst this may not fully reflect current practise/patterns, this timeframe was intentional as it will allow future interrogation of longer term outcome data beyond the first 1–2 years following transition from paediatric care, a body of work which is planned to take place as part of baseline comparative data for the ongoing Transition Compass randomised controlled trial [16]. These data were collected in a pre‐COVID cohort which will allow interrogation of this post‐transition outcome data according to the level of access to face to face healthcare in the initial stages of the post‐paediatric transition process. Since that time there has been a significant increase in telehealth reviews which facilitated transition support to a greater number of patients, and opportunities to run more joint clinics and case conferences for complex patients and to facilitate connection and handover across different health services. Whilst validation of the use of a complexity score to assess risk across the transition pathway is also required in this future work, at its most basic it provides a means to describe the level of multifaceted challenges faced by AYA. Complexity is potentially a measure by which resource allocation could be initially prioritised in terms of the intensity of support in the pre‐transfer preparation phase. It is also a necessary component of the information that is provided to receiving adult services such that appropriate supports for co‐morbidities beyond the AYA's principal medical condition can be considered and actioned. Consideration should also be given to how adult trainees and physicians can be best supported to identify and respond to the needs of AYAs entering the adult healthcare system. Future policy should consider the impact of complexity on transitioning AYAs so that a standardised and equitable approach to resourcing can be assured across states and territories. Those with higher needs should receive additional supports without neglecting the needs of the majority of AYAs undergoing healthcare transition.

Whilst success in terms of transition to adult care has yet to be fully defined, the clinical impacts of unsuccessful transition are significant and costly to both the individual (condition progression, financial toxicity) and healthcare system (disengagement from specialist teams with resultant reliance on crisis‐driven care). The extent to which pre‐transition supports can mitigate the risk of an adverse transition experience is unmeasured, but these data provide the opportunity to interrogate the relationship between pre‐transition status, transition service input and post‐transition health service utilisation outcomes. Siloed data based on single clinics or cohorts are unlikely to result in systemic change to the practise of transition to adult healthcare services. Data such as these provide a platform from which robust post‐transition outcome data can be generated which in turn will facilitate the definition of care standards and the development of robust policy.

## Author Contributions

M.W. designed the study, arranged appropriate institutional approval, determined the parameters of the EMR search, conducted the retrospective chart medical record review, drafted the manuscript and ensured that all author contributions are as stated. E.C. contributed to data concept and design and assisted with data interpretation. A.L. conducted the data cleaning. A.V. conducted the EMR data search and extraction. All authors contributed to the writing of the manuscript and reviewed the final submitted version. M.W. takes final responsibility for the integrity of this submission.

## Funding

The authors have nothing to report.

## Conflicts of Interest

Ms. Evelyn Culnane is Manager of the RCH Transition Support Service. The other authors declare no conflicts of interest.

## Data Availability

The data that support the findings of this study are available on request from the corresponding author. The data are not publicly available due to privacy or ethical restrictions.
